# Alcohol and Other Factors Affecting Osteoporosis Risk in Women

**Published:** 2002

**Authors:** H. Wayne Sampson

**Affiliations:** H. Wayne Sampson, Ph.D., is a professor of human anatomy and medical neurobiology and nutrition at Texas A&M University System Health Science Center, College of Medicine, College Station, Texas

**Keywords:** osteoporosis, bone mass density, risk factors, female, AODE (alcohol and other drug effects), alcoholic beverage, tobacco in any form, lifestyle, physical exercise, obesity, nutrition, estrogens, hormone therapy, literature review

## Abstract

By about age 35, people reach their peak bone mass. Women lose bone mass slowly after that point until a few years after menopause, when bone mass is lost very rapidly. For middle-aged and older women, healthy bones depend on the development, during younger years, of a strong bone structure and an adequate peak bone mass. There is tenuous evidence that moderate alcohol consumption may protect bone. But human and animal studies clearly indicate that chronic heavy drinking, particularly during adolescence and the young adult years, can dramatically compromise bone quality and may increase osteoporosis risk. Further, research indicates that the effects of heavy alcohol use on bone cannot be reversed, even if alcohol consumption is terminated. Research suggests that in addition to alcohol, other lifestyle factors—such as tobacco use, nutrition, weight-bearing exercise, increased body weight, and hormone replacement therapy—affect bone development and osteoporosis risk in women. However, there has been little examination of how alcohol interacts with these factors to influence bone health.

Osteoporosis is a skeletal disorder characterized by low bone mass, increased bone fragility, and susceptibility to fracture (see figure 1).^1^ Approximately one in two women and one in eight men over age 50 will have an osteoporosis-related fracture in their lifetime, and these fractures account for approximately $14 billion in direct medical costs (National Institutes of Health 1999).

At approximately age 35, people reach their “peak bone mass”—the point at which their bones are as dense, or strong, as they will become (Edelson and Kleerekoper 1995). After age 35, women lose 0.5 percent to 1 percent of their bone mass each year. At menopause, when the ovaries stop producing estrogen, the rate of bone loss increases, in the absence of estrogen replacement therapy, from 3 percent to 7 percent per year, building to 15 percent to 35 percent loss in bone mass in the first 5 years after menopause (Bonnick 1994).

For middle-aged and older adults to have healthy bones, they need to have developed a strong bone structure and an adequate peak bone mass during their younger years. Bone structure and peak bone mass are greatly affected by lifestyle factors, including alcohol use, especially during the adolescent and young adult years (see figure 2). This article reviews research on how alcohol use and other factors affect bone health and osteoporosis risk in women.

## Moderate Drinking

The effect of moderate* alcohol use on bone health and osteoporosis risk is unclear. A few epidemiological studies in humans have indicated that moderate alcohol consumption may be associated with decreased fracture risk in postmenopausal women (Hansen et al. 1991; Felson et al. 1995). One large study (Diaz et al. 1997) found that women age 65 and over who consumed alcohol on more than 5 days per week had a significantly reduced risk of vertebral deformity^2^ compared with those who consumed alcohol less than once per week.

**Figure 2:**
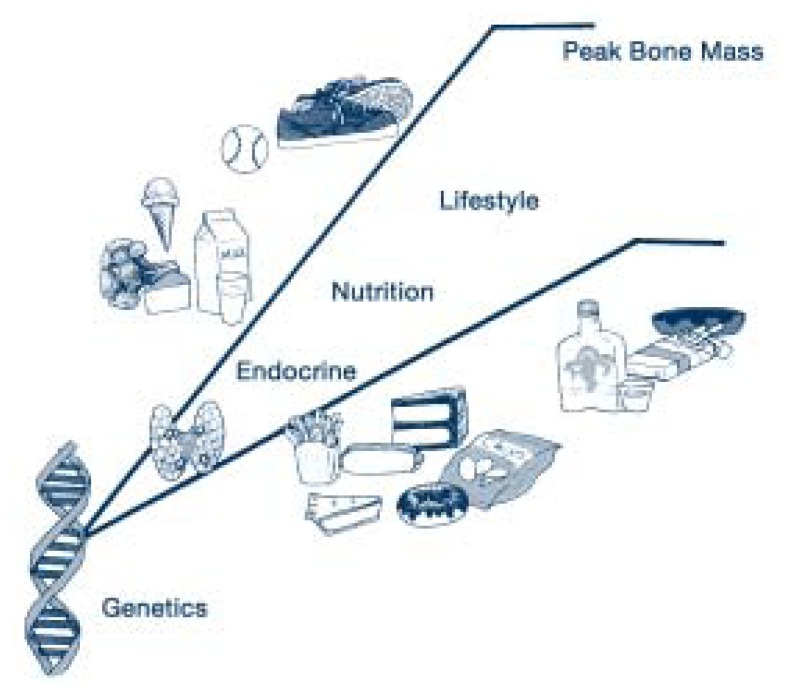
Peak bone mass is affected not only by genetic and other biological factors but also by lifestyle variables such as nutrition, exercise, tobacco use, and drinking. In this figure, factors along the steeper line contribute to higher peak bone mass. SOURCE: Amanda Arnold.

This apparent beneficial effect of moderate drinking on bone health has not been found in animal studies, which can control for the amount of alcohol consumed as well as for other lifestyle factors (see figure 2). For example, Sampson and Shipley (1997) gave ovariectomized and sham animals (animals in which abdominal surgery was performed but the ovaries were not removed) 0.38 g/kg of alcohol a day for 6 weeks (the equivalent of two glasses of wine per day, containing 12 g of alcohol per glass, for an average 63-kg woman). Removal of the ovaries led to decreased bone density and bone volume compared with control animals, but comparisons with animals that were not fed alcohol showed that these changes were not significantly altered by alcohol consumption. In contrast, in a study of rats administered alcohol for 4 months, Turner and colleagues (2001) reported a decrease in the replacement of old bone with new bone tissue (i.e., bone turnover) following moderate alcohol consumption. These studies found no beneficial effect of moderate alcohol use on bone quality.

## Chronic Heavy Drinking

### Effects of Alcohol on Growing Bone

Almost all epidemiological studies of alcohol use and human bone health indicate that chronic heavy alcohol consumption, particularly during adolescence and young adulthood, can dramatically affect bone health and may increase the risk of developing osteoporosis later. Although alcohol appears to have an effect on bone-forming cells (i.e., osteoblasts), slowing bone turnover, the specific mechanisms by which alcohol affects bone are poorly understood.

**Figure 3:**
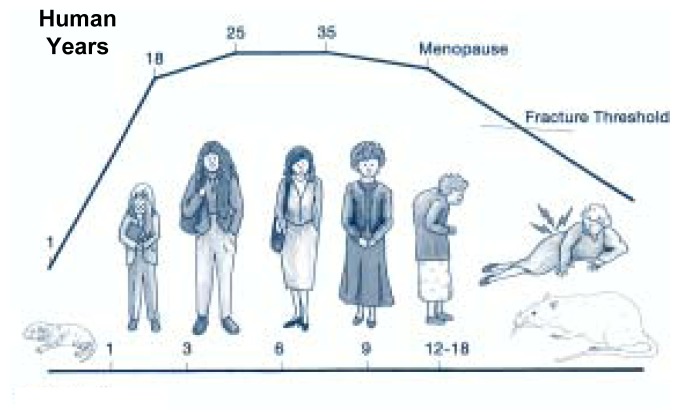
Comparison of rat and human ages.

Studies in female animals have also demonstrated unequivocally that early chronic alcohol consumption compromises bone health, including decrements in bone length, dry weight (weight of the bone with the water removed), and mineral content. Research has shown that young, actively growing rats chronically consuming alcohol had reduced femur lengths when compared with pair-fed control rats until they were approximately 9 months of age (see figure 3 for a comparison of rat and human ages). Eventually, the femurs of alcohol-fed animals caught up with the growth in length of animals in the control group (Sampson et al. 1997; Hogan et al. 1997).

This ability of the femur to make up for lost time, however, did not extend to all measures of bone health: Relative to control animals, alcohol-fed animals’ bone density was significantly reduced and remained so throughout the animals’ lives (Hogan et al. 1997).

In further examinations of these same animals, computer analyses of microscope slides of the upper tibia revealed greatly reduced bone volume in alcohol-fed rats compared with control rats (Sampson et al. 1997). In particular, the analyses showed a reduction in the number of thin plates (*trabeculae*) that form the soft, inner part of the bone. Further, after the animals had stopped growing, the overall thickness of the inner (cancellous) bone was also reduced in alcohol-fed rats compared with rats in a control group.

Additional evidence that alcohol causes bone-growth deficiencies in actively growing animals is provided by studies of the developing tissue, known as growth plates, near the ends of long bones (Sampson et al. 1997). These studies revealed that alcohol severely slowed the proliferation of cartilage cells, important precursors to bone development, and arrested longitudinal bone growth.

Studies of rats fed alcohol from 1 month of age throughout their lives indicate that alcohol-induced bone deficiencies may stem from a lag in growth, rather than from a loss of bone content (Sampson 1998). That is, the animals may not be losing bone per se, but they may not be growing and maturing as they should. This conclusion is supported by measurements of blood levels of a hormone, known as insulin-like growth factor 1 (IGF–1), that helps maintain bone density. In both groups of rats, IGF–1 values were greatest in younger animals and diminished until the animals stopped growing at 9 months old. Alcohol greatly reduced initial IGF–1 values; however, the magnitude of this reduction decreased with age, so that at 9 months, alcohol-fed rats’ IGF–1 values were similar to those of control animals. These findings lend support to the idea that during the younger years alcohol’s effect may be on growth more than on bone itself.

Finally, in these young animals, chronic alcohol exposure also compromised the bones’ mechanical properties, including their elasticity, stiffness, load-carrying capacity, and toughness (i.e., amount of deformation before breaking) (Hogan et al. 1997). It appears that bone’s cortical area (i.e., the tubular, mid-shaft part of the bone) and its shape in cross-section were unaffected by alcohol exposure. Over the long term, alcohol-fed animals seemed to adapt, at least partially, to these reductions in tissue quality and strength by producing generally larger bones with thinner cortical walls (Sampson 1998).

Nevertheless, the effects of alcohol consumption on bone could not be reversed, regardless of whether alcohol consumption continued or was terminated (Sampson et al. 1997; Hogan et al. 1997). The mechanical integrity of the alcohol-fed animals’ bones—the source of their strength—did not “catch up” with control animals.

### Effects of Alcohol on Adult Bone

Although alcohol’s damaging effects on bone are most striking in young people, research has shown that women between the ages of 67 and 90 who consumed an average of more than 3 ounces of alcohol per day (the equivalent of six drinks) had greater bone loss than women who had minimal alcohol intake (Hannan et al. 2000). (For information on how light-to-moderate drinking affects bone health in older women, see the article in this issue by Register and colleagues.) In addition to such research in human adults, studies of animals that began consuming alcohol as elderly animals also revealed deficiencies in bone volume and density (Hogan et al. 2001). These studies in adult animals agreed with cell-culture studies, suggesting that in these adult animals, alcohol consumption has greater deleterious effects on bone formation than on the breakdown (i.e., resorption) of old bone.

## Other Risk Factors for Osteoporosis

In addition to alcohol use, lifestyle factors such as tobacco use, exercise and body weight, nutrition, and hormone replacement appear to play a role in bone health and osteoporosis risk, although the magnitude of these roles is not well understood. Brief summaries of research on such osteoporosis risk factors are provided below, followed by a discussion of how alcohol might interact with each factor. However, as these discussions underscore, in most instances, there has been very little research addressing their association with alcohol’s effects.

### Tobacco Use

Many—although not all—studies have shown a link between tobacco use and decreased bone health. Heavy smoking has long been associated with greater risk for osteoporosis (Daniell 1972); a higher incidence of bone fractures, lower bone density, and fewer teeth (Johnston 1994); a dramatic decrease in the mineralization of bones in the hip, hand, forearm, and heel; decreased bone healing (Hollinger et al. 1999); and a decrease in new bone formation (Yuhara et al. 1999; Fang et al. 1991). Studies have demonstrated a causal link between heavy smoking and decreased bone mass (McCulloch et al. 1991; Friedl et al. 1992), whereas moderate or light smoking appears not to cause such harm (Daniel et al. 1992). Research with premenopausal female twins revealed that smokers had markedly lower bone densities than did their nonsmoking twins. Finally, some researchers have reported that among postmenopausal women, smokers lose cortical bone (i.e., tubular, mid-shaft bone) about 50 percent faster than do nonsmokers; however, the causes of this increased rate of bone loss are unclear.

Some recent research, however, has been more equivocal. Hannan and colleagues (2000) found that although older men who were current smokers lost more bone mineral density than did men who never smoked, there was no such difference between female smokers and nonsmokers. Likewise, recent research in animals (Syversen et al. 1999; Iwaniec et al. 2000) has failed to confirm the results of earlier studies that had reported a decrease in bone mineral density following smoking (Hollo et al. 1979) or nicotine exposure (Broulik and Jaráb 1993).

Despite uncertainty over the role of tobacco use in bone health, researchers have suggested several mechanisms by which smoking may affect osteoporosis risk. In postmenopausal women, smoking may speed the breakdown (metabolism) of estrogen, resulting in lower estrogen levels and increased bone loss and risk of fracture (Kiel et al. 1992). Other suggestions for the mechanism of smoking’s effect on bone include smokers’ lower body weight, decreased physical activity, decreased absorption of calcium, increased alcohol intake (see the next section) and other nutritional deficiencies, resistance to the hormone calcitonin (which suppresses bone resorption), and direct effects of tobacco on bone cells.

#### Alcohol and Tobacco Use

People who drink alcohol are 75 percent more likely to smoke than are nondrinkers, and smokers are 86 percent more likely to drink than are nonsmokers (Shiffman and Balabanis 1995). One activity attenuates the impact of the other—for example, smoking appears to slow the release of alcohol from the stomach, causing more alcohol to be broken down in the stomach and less to be absorbed into the circulation (Chen et al. 2001). By lowering blood alcohol concentrations in this way, smoking allows one to drink more before getting drunk. Although there has been little research addressing this issue, it seems reasonable that anything that increases alcohol consumption (as smoking does) might be detrimental to bone physiology. The only investigation of the combined effects of alcohol and tobacco on bone health, an epidemiological study by Deng and colleagues (2000), found no effect on bone mineral density as a result of drinking or smoking, but did find a significant effect in participants who both smoked and drank. However, as this research is in its infancy, these findings should be considered preliminary.

### Exercise and Obesity

Because the major determinant of whether or not a woman develops osteoporosis is her peak bone mass and her rate of bone loss, it is of interest to know whether exercise can influence either of these two factors (Stevenson et al. 1990). Although the mechanisms are not fully understood, mechanical stress—such as that imposed by muscle contraction and weight-bearing exercise—increases bone density (Marcus and Kiratli 1998; Snow et al. 1996). Bone that is immobilized and has no weight-bearing function, as occurs in space flight, spinal cord injury, or prolonged bed rest, tends to lose mass rapidly (Turner 2000).

Only a few studies have focused on the effect of exercise and bone mass in osteoporotic patients. This research found that patients who exercised showed an improvement in bone mineral density by 1 percent over time and by 2 to 3 percent above nonexercising control subjects (although the greatest benefit of exercise was to increase participants’ functional capacity and decrease the incidence of falls) (Marcus and Kiratli 1998; Millard 1996).

In addition, carrying extra body weight amounts to weight-bearing exercise, and thus, like weight-bearing exercise, obesity is also associated with both reduced risk and lessened severity of osteoporosis. In fact, there has been some suggestion that the association between smoking and osteoporosis may derive, at least in part, from the fact that smokers tend to be less obese than nonsmokers (Broulik and Kapitola 1993). Research shows that obese women lose relatively little bone at menopause, whereas thin women tend to have a greater risk for osteoporotic fractures (Broulik and Kapitola 1993). And in one study of older women, those in the lower weight quartiles and those who lost 5 percent or more of their weight during the study had significantly more bone loss than did those who were heavier or who did not lose significant weight during the study (Hannan et al. 2000).

The reasons for the link between obesity and lower osteoporosis risk are not fully understood. Some experts postulate that estrogen produced or stored in fat tissue might attenuate bone loss. In addition, having extra body weight means that most movement is “weight bearing,” and thus obesity is a powerful determinant of bone mass (Heany 1995).

#### Exercise, Obesity, and Alcohol

There has been little research on how alcohol consumption and exercise or weight might together influence osteoporosis risk. Preliminary studies of exercising, alcohol-fed animals have demonstrated that exercise did not mitigate any of the negative effects of alcohol (Reed et al. 2002).

### Nutrition

Bone requires many nutrients to develop and remain healthy, including calcium; phosphorus; zinc; manganese; copper; vitamins D, K, C, and A; and protein. During growth, it is especially important for people to take in enough calcium to build as high a peak bone mass as is genetically possible for them—a window of opportunity that remains at least partially open until women are in their thirties (Heaney 1995). Especially for pregnant and lactating mothers, it is necessary to replace the calcium lost daily through the kidneys, the intestine, and through sweat. When sufficient calcium is not brought in through the diet, it is removed from bone.

Vitamin D is also crucial to bone health, as it plays a major role in calcium absorption. Many people do not produce sufficient vitamin D through exposure to sunlight (because of variations in sun exposure, skin pigmentation, and aging-related decreases in the ability of skin to make the vitamin). For this reason, it is important for such people to eat foods that are rich in vitamin D, such as milk and other fortified foods and fish oils. Nutrition is also important to bone health in that it contributes to body weight; as discussed above, carrying increased body weight, by acting as weight-bearing exercise, helps build bone mass (Heaney 1995).

#### Nutrition and Alcohol

As is the case for other lifestyle factors that affect osteoporosis risk, little research has addressed how alcohol might interact with other aspects of nutrition to influence bone health. It is known, however, that severe alcoholics are usually malnourished, a condition that exacerbates alcohol-induced vitamin D deficiencies, and thus impairs bone health by impairing calcium absorption.

### Estrogen and Hormone Replacement

Undoubtedly the greatest risk factor for the development of osteoporosis in women is menopause, when estrogen levels drop precipitously. Normally, the breakdown of old, worn bone is balanced by formation of new bone. It is not known how estrogen normally regulates this balance of bone remodeling. What is clear is that when estrogen declines dramatically, as it does during menopause, the cells that are responsible for breaking down old bone (osteoclasts) live longer. Osteoclasts’ longer life span gives them increased capacity to break down bone, disrupting the balance between bone resorption and new bone formation and resulting in bone loss.

Recent research has shown that postmenopausal hormone replacement therapy greatly protects against loss in bone density (Hannan et al. 2000) and reduces the risk of hip, spine, and other osteoporosis-related fractures.

#### Estrogen and Alcohol

There has been speculation that alcohol’s effect on bone is mediated by estrogen, but the evidence to support this idea is unclear. Some studies in humans have demonstrated what may have been an estrogen effect. In contrast to these findings, most studies in animals have seen no changes in estrogen, testosterone, or conversion of androgens to estrogen (a process known as aromatization) as a result of alcohol consumption. Some researchers believe that the effect seen in humans was a red wine antioxidant effect rather than an effect of alcohol itself (Purohit 1998).

## Summary

Human and animal studies clearly demonstrate that chronic, heavy alcohol consumption compromises bone health and increases the risk of osteoporosis. In particular, heavy alcohol use decreases bone density and weakens bones’ mechanical properties. These effects are particularly striking in young people (and animals), but chronic alcohol use in adulthood can also harm bone health. Further, animal studies suggest that bones do not overcome the damaging effects of early chronic alcohol exposure, even when alcohol use is discontinued.

The effect of moderate alcohol consumption on bone health is less clear. Some research in humans has indicated that moderate drinking may boost bone mass, whereas animal studies have contradicted that idea.

Research suggests that in addition to alcohol, other lifestyle factors, such as tobacco use and poor nutrition, might result in a lower peak bone mass and increase the likelihood of bone fractures. Other factors—such as weight-bearing exercise, increased body weight, and hormone replacement therapy—appear to have positive effects on bone development. To date, however, there has been little investigation into how alcohol interacts with these other factors to influence bone health and osteoporosis risk.

Current examinations of alcohol’s effects on bone health suggest numerous directions for further investigation. It is important to study the mechanism of alcohol’s effect on bone at many levels. Does alcohol work through growth factors, or does it affect osteoblasts directly in the young? How specifically does alcohol target osteoblasts? Does it act through receptors, signal transduction pathways, or through other mechanisms? Finally, additional research should examine whether alcohol’s negative effects on bone can be reversed.

## Figures and Tables

**Figure 1:**
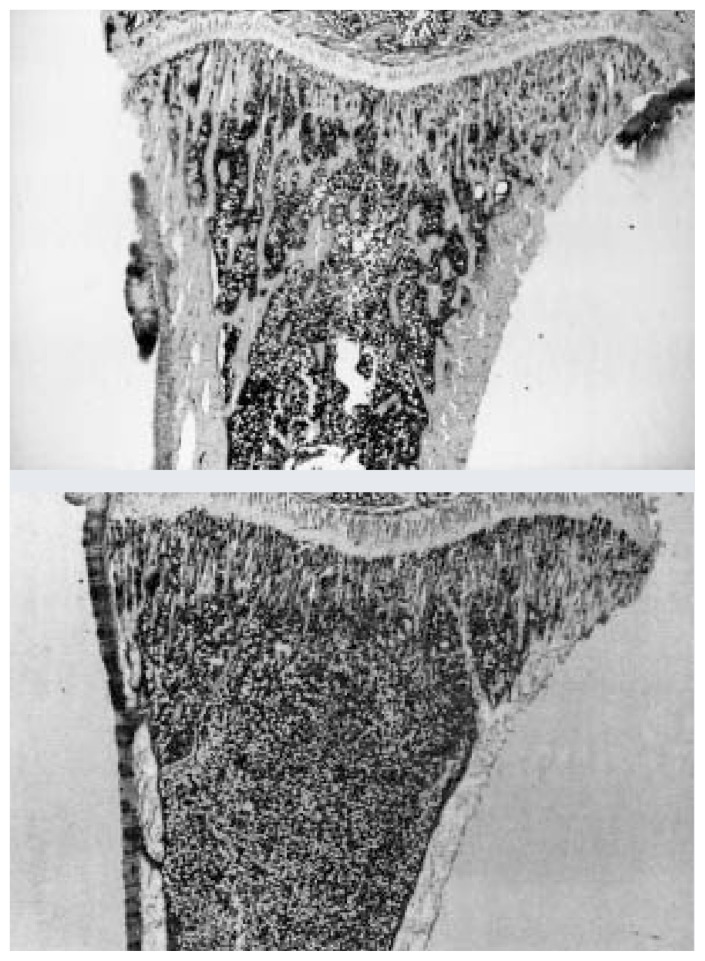
Normal bone (top) and bone from an alcohol-treated rat (bottom). Note that lighter-colored specules of bone are missing in the bottom image.

## Footnotes

* *Editor’s Note:* Definitions of moderate drinking vary. Federal guidelines consider moderate drinking to be no more than one drink per day for women and no more than two drinks per day for men (U.S. Department of Agriculture and the U.S. Department of Health and Human Services 1995).

1 Osteoporosis is classified as either primary or secondary depending on its underlying causes. Primary osteoporosis is subdivided into two types: Postmenopausal (Type I) osteoporosis occurs 15 to 20 years after menopause in females, following a decline in estrogen levels. Age-associated, or senile (Type II) osteoporosis occurs after approximately age 70 in both genders. In contrast to these two types of primary osteoporosis, secondary osteoporosis is the result of specific conditions such as disease, surgery, and drugs (Riggs and Melton 1988).

2 Crush fractures of the anterior vertebral body cause most women’s bone-related visits to the doctor.

## References

[b1-292-298] Bonnick SL (1994). The Osteoporosis Handbook.

[b2-292-298] Broulik PD, Jarab J (1993). The effect of chronic nicotine administration on bone mineral content in mice. Hormone and Metabolic Research.

[b3-292-298] Broulik PD, Kapitola J (1993). Interrelations between body weight, cigarette smoking and spine mineral density in osteoporotic Czech women. Endocrine Regulation.

[b4-292-298] Chen WJA, Parnell S, West JR (2001). Nicotine decreases blood alcohol concentration in neonatal rats. Alcoholism: Clinical and Experimental Research.

[b5-292-298] Daniel M, Martin AD, Drinkwater DT (1992). Cigarette smoking, steroid hormones, and bone mineral density in young women. Calcified Tissue International.

[b6-292-298] Daniell HW (1972). Osteoporosis and smoking. JAMA: Journal of the American Medical Association.

[b7-292-298] Deng HW, Chen WM, Conway T (2000). Determination of bone mineral density of the hip and spine in human pedigrees by genetic and life-style factors. Genetic Epidemiology.

[b8-292-298] Diaz NM, O’Neill TW, Silman AJ, the European Vertebral Osteoporosis Study Group (1997). Influence of alcohol consumption on the risk of vertebral deformity. Osteoporosis International.

[b9-292-298] Edelson GW, Kleerekoper M, Matkovic V (1995). Bone mass, bone loss, and fractures. Physical Medicine and Rehabilitation Clinics of North America.

[b10-292-298] Fang MA, Frost PJ, Iida-Klein A (1991). Effects of nicotine on cellular function in UMR 106-01 osteoblast-like cells. Bone.

[b11-292-298] Felson DT, Zhang YQ, Hannan MT (1995). Alcohol intake and bone mineral density in elderly men and women—The Framingham Study. American Journal of Epidemiology.

[b12-292-298] Friedl KE, Nuovo JA, Patience TH (1992). Factors associated with stress fracture in young army women: Indications for further research. Military Medicine.

[b13-292-298] Hannan MT, Felson DT, Hughes BD (2000). Risk factors for longitudinal bone loss in elderly men and women: The Framingham osteoporosis study. Journal of Bone and Mineral Research.

[b14-292-298] Hansen MA, Overgaard K, Riis BJ, Christiansen C (1991). Potential risk factors for development of postmenopausal osteoporosis— Examined over a 12-year period. Osteoporosis International.

[b15-292-298] Heany R, Kraft GH, Matkovic V (1995). Nutrition and bone mass. Physical Medicine and Rehabilitation Clinics of North America.

[b16-292-298] Hogan HA, Sampson HW, Cashier E (1997). Alcohol consumption by young actively growing rats: A study of cortical bone histomorphometry and mechanical properties. Alcoholism: Clinical and Experimental Research.

[b17-292-298] Hogan HA, Argueta F, Moe L (2001). Adult-onset alcohol consumption induces osteopenia in female rats. Alcoholism: Clinical and Experimental Research.

[b18-292-298] Hollinger JO, Schmitt JM, Hwang K (1999). Impact of nicotine on bone healing. Journal of Biomedical Materials Research.

[b19-292-298] Hollo I, Gergely I, Boross M (1979). Influence of heavy smoking upon the bone mineral content of the radius of the aged and effect of tobacco smoke on the sensitivity to calcitonin of rats. Aktuel Gerontologie.

[b20-292-298] Iwaniec UT, Fung YF, Cullen DM (2000). Effects of nicotine on bone and calciotropic hormones in growing female rats. Calcified Tissue International.

[b21-292-298] Johnston JD (1994). Smokers have less dense bones and fewer teeth. Journal of the Royal Society of Health.

[b22-292-298] Kiel DP, Baron JA, Anderson JJ (1992). Smoking eliminates the protective effect of oral estrogens on the risk for hip fracture among women. Annals of Internal Medicine.

[b23-292-298] Marcus R, Kiratli BJ, Stevenson JC, Lindsay R (1998). Physical activity and osteoporosis. Osteoporosis.

[b24-292-298] McCulloch RG, Whiting SJ, Bailey DA (1991). The effect of cigarette smoking on trabecular bone density in premenopausal women, aged 20–35 years. Canadian Journal of Public Health.

[b25-292-298] Millard PS, Rosen CJ (1996). Prevention of osteoporosis. Osteoporosis: Diagnosis and Therapeutic Principles.

[b26-292-298] National Institutes of Health Osteoporosis and Related Bone Diseases—National Resource Center (NIH ORBD NC) (1999). Osteoporosis Overview.

[b27-292-298] Purohit V (1998). Moderate alcohol consumption and estrogen levels in postmenopausal women: A review. Alcoholism: Clinical and Experimental Research.

[b28-292-298] Reed AH, McCarty HL, Evans GL (2002). Effects of chronic alcohol consumption and exercise on the skeleton of adult male rats. Alcoholism: Clinical and Experimental Research.

[b29-292-298] Riggs BL, Melton LJ (1988). Osteoporosis: Etiology, Diagnosis and Management.

[b30-292-298] Sampson HW (1998). The effect of alcohol consumption on adult and aged bone: A histomorphometric study of the rat animal model. Alcoholism: Clinical and Experimental Research.

[b31-292-298] Sampson HW, Shipley D (1997). Moderate alcohol consumption does not augment bone density in ovariectomized rats. Alcoholism: Clinical and Experimental Research.

[b32-292-298] Sampson HW, Chaffin C, Lange J (1997). Alcohol consumption by young actively growing rats. A histomorphometric study of cancellous bone. Alcoholism: Clinical and Experimental Research.

[b33-292-298] Shiffman S, Balabanis M, Fertig JB, Allen JP (1995). Associations between alcohol and tobacco. Alcohol and Tobacco: From Basic Science to Clinical Practice.

[b34-292-298] Snow CM, Shaw JM, Matkin CC, Marcus R, Feldman D, Kelsey J (1996). Physical activity and risk for osteoporosis. Osteoporosis.

[b35-292-298] Stevenson JC, Lees B, Fielding C, Smith R (1990). Exercise and the skeleton. Osteoporosis 1990.

[b36-292-298] Syversen U, Nordsletten L, Falch J (1999). Effect of lifelong nicotine inhalation on bone mass and mechanical properties in female rat femurs. Calcified Tissue International.

[b37-292-298] Turner RT (2000). What do we know about the effects of space flight on bone?. Journal of Applied Physiology.

[b38-292-298] Turner RT, Kidder LS, Kennedy A (2001). Moderate alcohol consumption suppresses bone turnover in adult female rats. Journal of Bone and Mineral Research.

[b39-292-298] U.S. Department of Agriculture (USDA) and the U.S. Department of Health and Human Services (1995). Nutrition and Your Health: Dietary Guidelines for Americans.

[b40-292-298] Yuhara S, Kasagi S, Inoue A (1999). Effects of nicotine on cultured cells suggest that it can influence the formation and resorption of bone. European Journal of Clinical Pharmacology.

